# Investigation of Kurdish students’ L2 motivational self-system and their motivational beliefs in high school

**DOI:** 10.3389/fpsyg.2022.974748

**Published:** 2022-10-25

**Authors:** Kameran Noori Abdullah, Özge Razi

**Affiliations:** ^1^School of Foreign Languages, Faculty of Education, Cyprus International University, Nicosia, Cyprus; ^2^Department of Foreign Languages Education, Faculty of Education, Cyprus International University, Education and Humanities Center, Nicosia, Turkey

**Keywords:** motivation, motivational beliefs, leadership, culture factors, level of motivation, type of motivation

## Abstract

This study aims to examine and compare female and male Kurdish EFL students’ level and type of motivation based on L2 motivational self-system components and to identify their dominant type of motivation. The participants of this study were 118 students (46 female, and 72 male) were randomly selected as the participants of this study from different cities of Erbil governorate in Kurdistan region of Iraq. A Learners questionnaire used following the application of semi-structured interview sessions with learners who participated in the study. The data obtained from the questionnaire were analyzed through SPSS software, and the interview results were interpreted and analyzed through content analysis. The findings of this study revealed that both male and female Kurdish EFL students generally have high levels of motivation and the attitudes of both groups of learners were found to be generally positive towards English language and its learning. The most frequent motivational strategies, ways of motivating and arranging for them a suitable culture and recommended by the teachers in this study are helpful, especially for teacher educators in teacher training programs, so that they can get aware of the most used strategies by Kurdish EFL teachers in high school to be the leadership for this purpose, and try to improve and correct them, and try to teach them the most effective strategies and ways of raising motivation in the students.

## Introduction

Every human being is born with the learning ability and the learning happens in every stage of our lives consciously or unconsciously according to our needs. However, considering the educational programs, like any other formal or specific programs, learning happens consciously and therefore it cannot effectively and successfully takes place if the students do not have enough motivation. In fact, conducting research on the issue of motivation and its related concepts can help us to find the answer to the question of why some students are eagerly involved the class activities, do their homework eagerly, and do much effort in learning, while some others avoid doing their class assignments and if they do the class activities or study, they do it reluctantly. More specifically, in the case of foreign language learning, the students need to find enough reasons and interest for learning the target language. Since their beliefs and attitudes towards learning a foreign language, and in general the extent of their motivation, is determiner of their language learning achievement, it is important to study about their level of language learning motivation, their dominant type of motivation, and the factors which play the most important role in motivating them. Accordingly, this study aims at investigating and comparing the extent of male and female Kurdish students’ motivation in learning the English language as a foreign language in their classes, to explore the relationship between L2 Motivational Self System components (i.e., ideal L2 self, ought-to L2 self, L2 learning experience) with their language learning motivation and their language proficiency; and also to identify the most frequent type of motivation they have in terms of integrative and instrumental, and their attitudes towards language learning in general. Accordingly, considering the theoretical framework, this study has been partly conducted based on the L2 motivational self-system model proposed by Dornyei (2005, 2009) which has combined the famous paradigms from the research areas of L2 motivation and motivational psychology such as the theory of possible selves that was introduced by Markus and Nurius (1986) and self-discrepancy theory proposed by Higgins (1987).

Conducting this study is very beneficial in providing useful information for the educational decision makers, authorities, teachers, and scholars in this field to answer such questions and look for appropriate strategies and plan effective programs to enhance the students’ learning motivation and in the case of our study, the students’ language learning motivation and attitudes.

While many studies have been conducted in the area of language learning motivation, these types of studies on the Kurdish students have been rarely found in the literature, which calls for more investigation in this context.

To date, many research works have been conducted in the domain of motivation and foreign language learning, most of which were carried out in recent years (e.g., Hsieh, 2008; Matsumoto, 2009; Hsieh and Kang, 2010; Carreira, 2011, 2012), however in the context of Kurdistan region of Iraq, very few studies have been found in the literature that addressed the issues of language learning motivation, motivational beliefs, motivational teaching strategy, and L2 motivational self-system all together (e.g., Hussein and Bajalani, 2019; Ali et al., 2020). The lack of a comprehensive study that encompasses these issues is really felt in the educational system of Iraqi Kurdistan. Therefore, the present study is considered important for studying both learners’ and teachers’ beliefs, opinions and attitudes towards motivation and also investigating the l2 motivational self-system components, types of motivation Kurdish EFL students mostly have regarding English language learning, which were not fully and comprehensively covered in the previous studies. Moreover, in this study for the first time in the context of Kurdistan of Iraq, the factor of gender will be considered in investigating the learner’s motivation and factors of gender and experience will be considered for investigating teachers’ motivational teaching strategies, which is important.

### Limitations of the study

Every research work comes with some limitations and similarly, this study also faced with few of them. One of the limitations that were faced in this research was due to some students’ unwillingness to participate in the study. In this regard, some of the students did not fill the questionnaires that were administered to them and did not turn them back and in case of interview sessions, majority of the participants did not permit the sound-recording, which could be helpful in giving more complete and accurate data. It must be declared that the data were collected during the limited time that the schools were opened in Kurdistan region of Iraq before they were closed again due to the COVID-19 virus pandemic, and all ethical considerations were observed during the data collection process.

## Term definition

### L2 motivational self-system

Dornyei (2009) attempted to apply the psychological theories of “possible selves” into L2 motivation theory which resulted in proposing a new framework called L2 motivational self-system. This framework is comprised of three main components: Ideal L2 self, Ought-to L2 self, L2 learning experience. Ideal L2-self concerns the L2-specific aspect of one’s “ideal self,” or an ideal mental vision that an L2 user aspires to be in the future that embodies private hopes and aspirations. It is considered as a powerful motivator in the learner that makes him to invest efforts to decrease the discrepancy between the actual self and the ideal self. Ought-to L2 Self refers to the features that one should possess to avoid probable negative consequences of perceived duties, obligations, or responsibilities. It is extrinsic in nature because it includes the wishes and expectations of significant others such as fulfillment of the expectation of a teacher or boss or attempts not to fail an exam, which therefore might have little resemblance to the individual’s own desires and wishes. L2 Learning Experience refers to the learners’ situated executive motivational reasons concerning the immediate learning environment and experience or their attitude toward L2 learning. Therefore, it can be affected by situation-specific motives such as the curriculum, teacher, the peer group, and the teaching materials.

### Literature review

Considering the type of motivation that students have regarding English language learning, Lucas (2010) found that intrinsic motivation was the most dominant type of motivation among the participants in his study, in learning the reading and speaking skills for gaining language learning achievement. In contrast to the finding of Lucas (2010), Rezaee et al. (2015) found that compared to high achieving students in learning English as a foreign language, less talented or low achieving students were significantly more extrinsically oriented and were more concerned. On the other hand, in the study by Zanghar (2012) and Kashefian-Naeeini et al. (2018), both instrumental and integrative types of motivation were observed among learners, however they showed that compared to integrative motivation, the students were more instrumentally motivated. In contrast to the findings of Zanghar (2012) and Kashefian-Naeeini et al. (2018) showed that the participants in his study mostly had integrative motivation than the instrumental type of motivation. Moreover, Ditual (2012) that had found high levels of motivation and positive attitudes towards language learning, among the participants in his study, also showed that they had both integrative and instrumental types of motivation.

Considering the learners’ motivation, in the studies by Al-Sohbani (2015), it was found that the EFL students had high levels of motivation regarding English language learning. Özütürk and Hürsen (2014) also revealed that in comparison to male students, female students showed significantly higher range of motivation towards learning English and in comparison to sophomores, fresh men students had higher level of English language learning motivation.

Regarding the factors affecting students’ motivation, Rostami et al. (2015) came to the conclusion that participants’ families and teachers play vital roles in encouraging and motivating the students to learn English language as a foreign language, and therefore, are considered as effective factors in this issue. Chang (2010) found that having group works in class and the presence of motivated group members positively affects the other students’ level of motivation, while having distracted and demotivated students in class, has negative impact on motivating the other students. Fatiha et al. (2014) also found that teaching methodology and teachers’ attitudes positively affect learner’s motivation and motivation influences the learners’ attitudes regarding foreign language learning while both are essential for an effective language learning. On the other hand, Abdullah and Al-Mofti (2017) found that the most effective factors which had impact on increasing the Kurdish EFL students’ level of motivation were external social intention, support, and goals. According to them, the outside social support was the most influential factor on Kurdish students’ motivation. And there was a significant correlation between the leaners’ goals and the social supports they received. To mention other factors, Ceylan (2016) proposed that according to teachers’ beliefs and opinions in his study, students’ self-confident is an essential factor since this factor is important for motivating them, therefore teachers are supposed to consider raising their students self-confidence to get the best results from them. Gharghani et al. (2019) instead found that factors including higher self-efficacy and using various cognitive strategies are helpful for students in motivating them and facilitating their learning of the target language. Unlike the previous findings, Yurtseven et al. (2015) considered enjoyable learning atmosphere as the most effective factor in motivating the students. Tilya (2012) also highlighted the importance of motivating students by teachers and its positive impacts on students’ academic achievements.

Regarding EFL students’ perceptions towards English language learning and the issues related to the foreign language learning (e.g., the target language itself, the culture related to that language, the teacher, etc.), we can briefly mention to the following studies and their findings that correspond to the findings of the present study:

Al-Sohbani (2015) indicated that most of Iranian students that participated in his study had favorable attitudes regarding both English language learning and English language instructor. Moreover, Özütürk and Hürsen (2014) found that Turkish students have positive attitudes towards English language learning. Similarly Kashefian-Naeeini et al. (2018) showed that the students in their research had significantly high levels of positive and favorable attitudes regarding English language learning. Likewise, in the context of Kurdistan region of Iraq, Hussein and Bajalani (2019) indicated that the Kurdish EFL learners had a positive attitude regarding the motivation’s impact on the learner’s autonomy; however, although the participants showed high levels of interest to turn in to autonomous learners, they did not apply their positive perceptions towards this issue in practice.

Regarding the L2 motivational self-system, The results of the study by Magid (2014) showed the effectiveness of L2 motivational self-system on motivating the students to learn English. Similarly, Khan (2015) found that while ought to L2 self has only a key impact on students’ motivational level in terms of their English language learning efforts, L2 Ideal self has a high level of impact on both English language learning motivation and the participants’ English language learning achievement.

The results of the study by Papi and Teimouri (2012) showed that the students improve the variables such as attitudes regarding L2 community and culture, the, instrumentality promotion, L2 learning experience, and ideal L2 self, which are known as promotion-focus variables; in contrast the variables including instrumentality-prevention, the family influence, and ought-to L2 Self, declined with the raise of age, during their education before entering the university.

Kim and Kim (2014) found that English language learning was more influenced by Ought-to L2 self and ideal L2 self, compared to the self-regulation skills. Zoabi (2012) found that there is a positive relationship between the students’ self-image and their learning motivation.

Rajab et al. (2012) found that there is a significant relationship between the learners’ ideal L2 self and their intended attempts to learn English. In line with this finding, Moskovsky et al. (2016) found that L2 motivational self-system components were a good predictor of the students’ achievement and learning efforts. However, Yaghubnezhad et al. (2017) reported different representations of these three components of L2 motivational self-system during three timescales of their research. Sherafati and Ghafournia (2019) also found a positive relationship between reading comprehension skill and L2 motivational self-system.

In contrast to the above findings, in the studies by Papi and Abdollahzadeh (2012) and Mac Intyre and Serroul (2015), the ideal L2 self was not found to be predictable of the students’ actual classroom performance and they did not find it effective in motivating the students. Similarly, Moskovsky et al. (2016) found a weak and uncertain relationship between the self-guides and language achievement and found that the ideal L2 self was a negative predictor of language proficiency in some cases.

### Research questions

Generally, this study intends to answer the following 2 main questions:

1. Is there any difference between male and female Kurdish students’ beliefs and perceptions towards their own motivational level, type of motivation, and the effectiveness of the motivational strategies used by their teachers?
2. Is there any difference between the L2 motivational system components (i.e., ideal L2 self, ought-to L2 self, L2 learning experience) which mostly affect male and female Kurdish students’ motivation and language proficiency?

## Methodology

### Participants

The participants of this study were 118 students, including 46 female and 72 male Kurdish students. The detailed description of the participants is illustrated in Table 1. In this regard, the sample of this study was randomly selected from all EFL students that attended English language classes at high schools in the different cities of Erbil (the biggest governorate of the Kurdistan region of Iraq).

**Table 1:** Detailed description of the students who participated in this study.

Learners sample		
Gender	Female	46
	Male	72
Language	The native language of all participants was Kurdish	
Age	All learner participants aged between 17 and 18	
Total Number		118

Moreover, 36 students who showed consent and willingness to be interviewed were selected to participate in the interview sessions in order to elaborate on their thoughts and perceptions.

### Instruments

#### Questionnaire

As a quantitative data collection tool, a contextually adapted and modified version of a questionnaire that was originally developed by Dornyei et al. (2005) was used in this study (see Supplementary Appendix). This questionnaire included 41 items, it must be asserted that the items used in this questionnaire are divided into 7 groups that each group represents a specific factor.

In designing this questionnaire, a 6-point Likert scale was used for the items, where considering the statement-type items, the participants were supposed to choose a number for their answers from 1 to 6 (1 = Strongly Disagree, 2 = Disagree, 3 = Slightly Disagree, 4 = Slightly Agree, 5 = Agree, 6 = Strongly Agree).

#### Interview

In this study, the qualitative data obtained from interview sessions for learners were analyzed descriptively through content analysis method. The researcher partly designed the questions raised in the interview sessions according to the aims of the study, and the other part was based upon the items they had responded to before in the questionnaires. In this regard, the participants’ responses were typed exactly the same way as they were uttered, so that the data would be kept authentic and without any modification or correction. Then all the participants’ answers were typed and listed under each question that was asked to organize the data and to facilitate the data interpretation process. After that, the answers were all reviewed by the researcher through document and content analysis, and then through open-coding strategy, the whole data were analyzed and interpreted.

### Procedure

The learner motivation questionnaire was administered to the students at the end of the academic semester, so that the students could better answer the questionnaire for they had experienced the English language class for enough time with their teacher. The questionnaire was also administered in the presence of the researcher, so that in case of any ambiguity the researcher could answer the participants’ questions and solve their possible problems in understanding the questionnaire. Moreover, the researcher translated the items which seemed difficult to understand for the students, so that they could answer without worries of not understanding, and so their responses would be more accurate.

After collecting data through questionnaire, the volunteered participants were selected to be interviewed. The researcher took the notes of the students’ responses for most of the participants did not agree their voice to be sound recorded. So the researcher also used an assistant in order not to miss anything from the participants’ responses to the interview questions.

### Ethical considerations

Regarding the ethical considerations, before administrating the questionnaires, the nature and goals of the study were briefly explained to the students in a way that they do not affect the answers they provide and hence the reliability of the data which was going to be collected. The participants were also assured of the confidentiality of the data they would provide and they were asked to return the questionnaires anonymously.

The same ethical issues were also considered in data collection procedure for the interview. Before conducting the interview, the participants’ permission was asked to sound record the interview sessions to be used for the easier data analysis, and therefore for those who felt uncomfortable, the interview was conducted without sound-recording, and only by note-taking. Unfortunately, few of the participants agreed on sound recording the interviews, and the others were note-taken.

It must be asserted that all participants were assured of the confidentiality of the data they provided and that the researcher is the only one who will have access to the collected data and specially the sound-recorded data.

#### Inferential and descriptive statistics of learners’ questionnaire

The participants’ responses to the questionnaire were analyzed through the frequency analysis method which shows the distribution of the participant’s responses to each item in the questionnaire and was interpreted based on each of these 7 mentioned scales or categories that this questionnaire aimed to assess. First, the results of the participant’s responses will be represented as a whole for all seven scales. Then the responses of the participants in the groups of females and males will be represented separately and compared.

## Data analysis and results

To analyze the data collected through questionnaire, SPSS software was used and the results were represented through tables and diagrams, using frequency analysis and descriptive statistics. On the other hand, content analysis method and open-coding strategy was used to descriptively analyze and interpret the data obtained from interview.

### Inferential and descriptive statistics of learners’ questionnaire

The participants’ responses to the questionnaire were analyzed through the frequency analysis method which shows the distribution of the participants’ responses to each item in the questionnaire, and were interpreted based on each of these 7 mentioned scales or categories that this questionnaire aimed to assess. First the results of the participants’ responses will be represented as a whole for all seven scales. Then the responses of the participants in the groups of female and male will be represented separately and compared as it can be seen in Table 2.

**Table 2:** Comparison of the mean of students’ responses to the 7 scales of questionnaire with the criterion value.

Scales	Test value = 3.5			
	Mean	*t*	df	Sig. (2-tailed)
Criterion measures	3.6638	2.472	117	0.015
Ideal L2 self	4.0198	5.799	117	0.000
Ought-to L2 self	4.2105	9.968	117	0.000
Family influence	4.4972	14.145	117	0.000
Attitudes to learning English	4.3404	14.375	117	0.000
Fear of assimilation	2.4915	−15.907	117	0.000
English anxiety	3.9958	4.702	117	0.000

### Overall analysis of participants’ responses to each of the 7 scales of the questionnaire

**Diagram 1:**
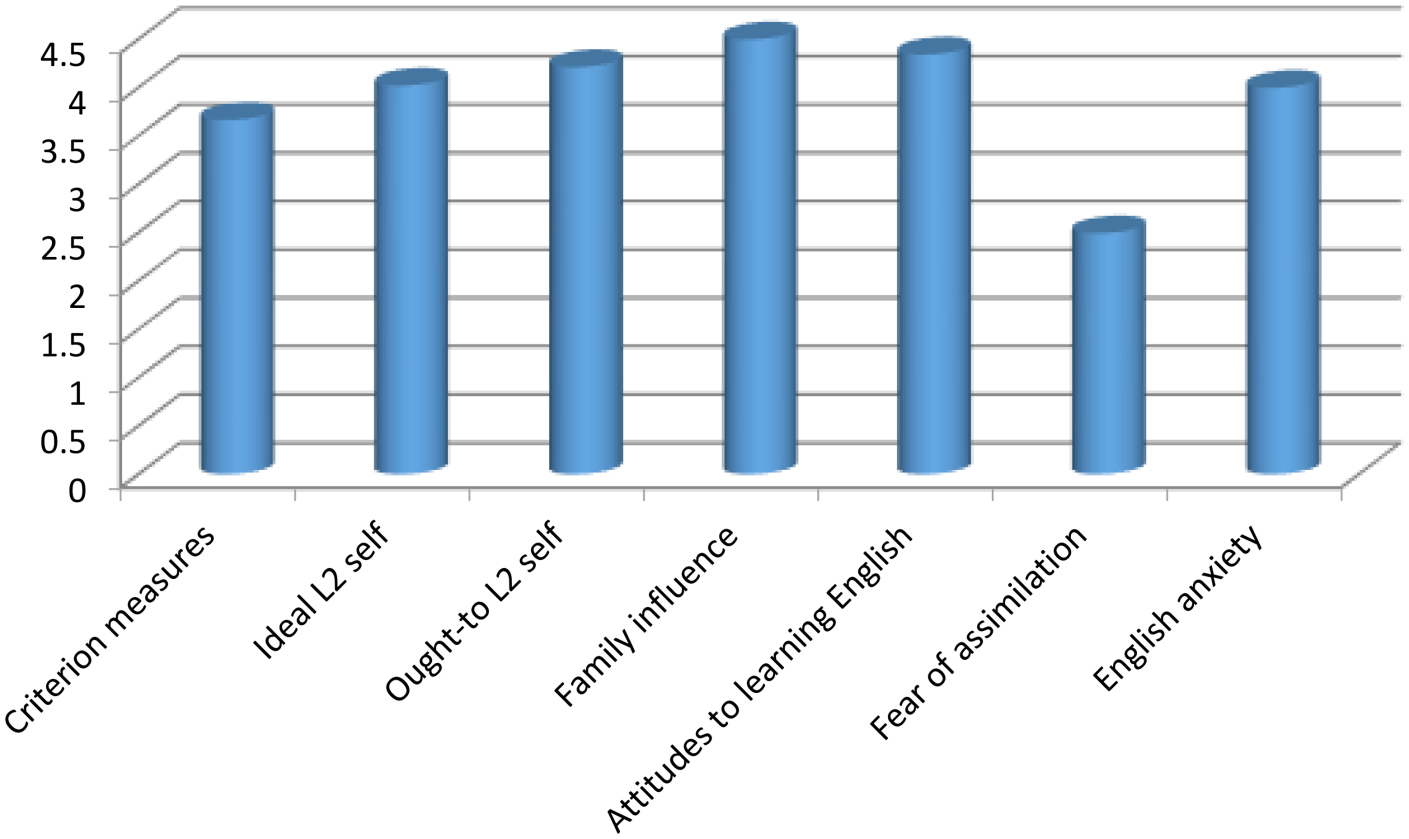
Analysis of the level of 7 factors (scales) that affect students’ motivation, based on their responses.

According to Diagram 1, analyzing the level of 7 scales under study based on the students’ responses shows that scales 1 to 5 (Criterion measures, Ideal L2 self, Ought-to L2 self, Family influence, Attitudes to learning English), and 7 (English anxiety) were above the average level and hence had a higher impact on the students’ motivation. However, factor 6 (Fear of assimilation) which was below average level as shown in the diagram is also considered to have a high impact on students’ motivation, due to the types of items raised in this scale. A comparison of all 7 factors together is shown in Table 3.

**Table 3:** Comparison of students’ responses to all scales of the questionnaire.

Tests of within-subjects effects							
Measure: MEASURE_1							
Source		Type III Sum of Squares	df	Mean Square	F	Sig.	Partial Eta Squared
Factors		319.673	4.216	75.829	98.537	0.000	0.457
Error (factor1)		379.570	493.241	0.770			

According to the obtained results, as shown in Table 3, it can be observed that there is a significant difference in the average level of 7 scales so that some are located at a higher level and some are at a lower level. It can be seen that scale 6 is at the lowest level, followed by scales 1, 7, 2, 3, 5, and finally factor 4. In fact, scale 6 has been different from everyone else, which is statistically right but due to the nature of the items in this scale, this difference is not counted.

#### Analysis and comparison of male and female participants’ responses to each of the 7 scales of questionnaire Table 4. Male vs. female participants’ distribution of responses to each of the 7 categories of the questionnaire (see Supplementary Appendix)

**Table 4:** Male vs. female participants’ distribution of responses to each of the 7 categories of the questionnaire.

Name of scales	Gender				Compare		
	Male (N = 72)		Female (N = 46)				
	Mean	Sd	Mean	Sd	*t*-test	df	Sig
Criterion measures	3.76	0.67	3.51	0.78	1.864	116	0.065
Ideal L2 self	4.04	1.02	3.98	0.91	0.337	116	0.737
Ought-to L2 self	4.19	0.74	4.24	0.83	−0.361	116	0.719
Family influence	4.46	0.73	4.56	0.82	−0.688	116	0.493
Attitudes to learning English	4.42	0.65	4.22	0.60	1.644	116	0.103
Fear of assimilation	2.41	0.70	2.63	0.65	−1.710	116	0.090
English anxiety	3.93	1.19	4.09	1.08	−0.745	116	0.458

**Diagram 2:**
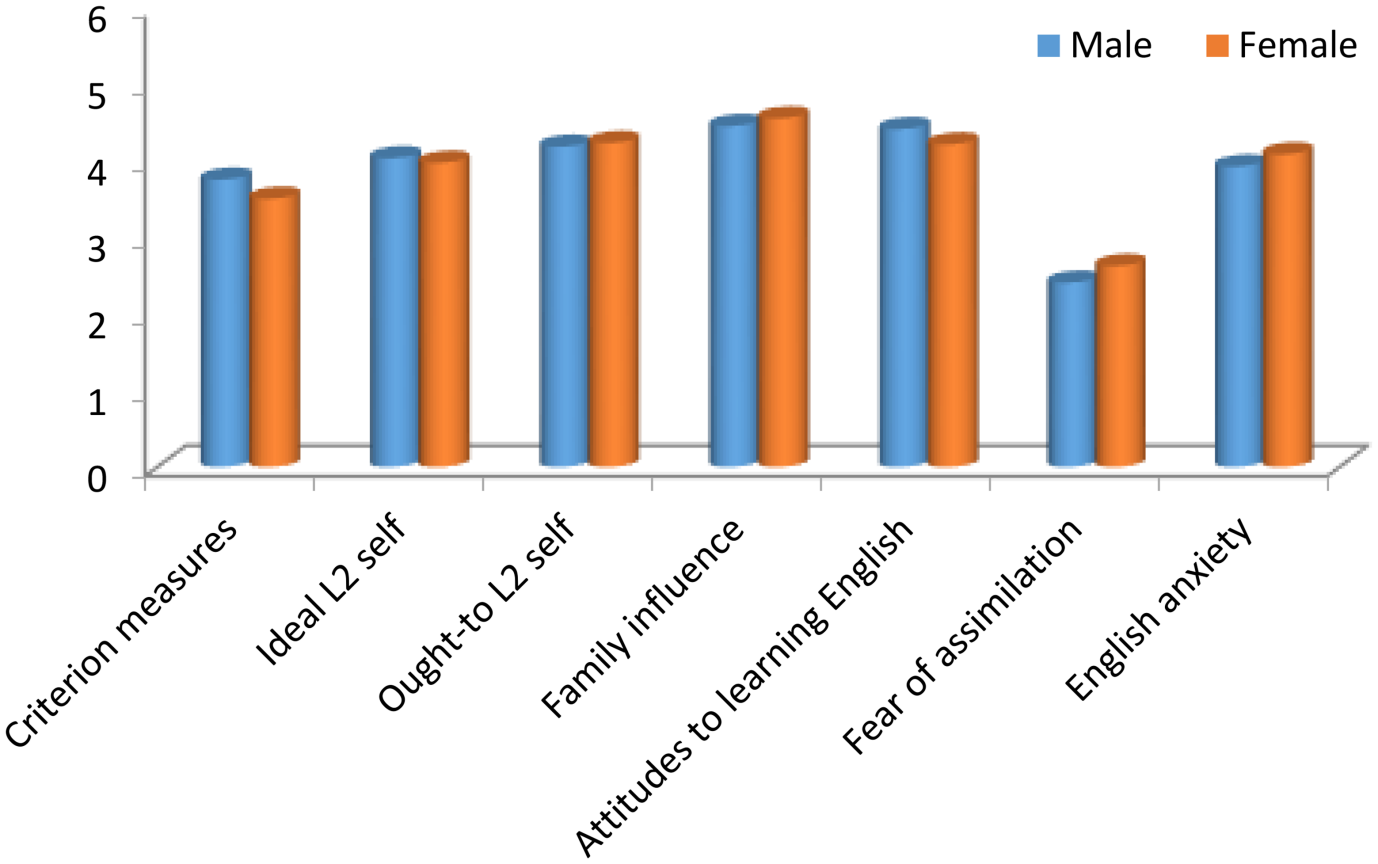
Comparison of the male and female students’ responses to each of the 7 scales.

According to the results of comparing the male and female students’ responses to the questionnaire scales, as shown in Table 4 and Diagram 2, it can be seen that there is no significant difference between the two groups of students in their answers to the different scales of the questionnaire.

### Learners’ interview results

Considering the results of learners’ interviews and based on the factor of gender, it is noteworthy that generally no significant difference had been observed between male and female students’ general beliefs and attitudes in the interview section. Hence, since most of the participants’ responses were almost similar to each other, we raised a few examples of responses in elaborating on learners’ responses to the interview questions.

**Learners’ responses to question 1** (How much do you consider yourself motivated or demotivated regarding English language learning?)

The majority of the participants (79.6%) considered themselves motivated in learning the English language, while the rest of them considered themselves demotivated in this respect. To mention some of the responses and the participants’ elaboration on their answers we can refer to the following responses which belonged to the students who considered themselves motivated:

“Motivated. Yeah, I am motivated enough to eagerly learn this language. You know, I like to travel abroad and visit different countries and meet people from different cultures and of course languages. I feel learning about different languages is amazing, but I prefer English the most and I choose it to learn and I do it eagerly because I think no matter where to travel, wherever I go, there are people who can understand English and I can use it to communicate different people. That’s why I feel very motivated in learning this language.”

**Learners’ responses to question 2** (What factors do you think were effective in motivating or demotivating you for learning the English language?)

The most frequent effective factors that the participants mentioned, respectively, included teacher (90.6%), the better job opportunities in the future (82.8%), more future academic success (75%), educational setting (60.9%), class atmosphere (87.5%), learning facilities (70.3%), method or ways of teaching by teachers (93.7%).

To mention some of the responses and the participants’ elaboration on their answers we can refer to the following responses:

“I think my teacher played the most important role in demotivating me in learning English. I think teachers’ teaching practices, strategies, and behaviors are very effective in motivating or demotivating the students. My teacher’s way of teaching for example is not attractive and his behavior is so cold.

“I think many factors are involved including families, society, and teachers mostly, and at the end, school and educational setting. I think they all are important factors. They can all have an impact on the students’ motivation or demotivation.”

**Learners’ responses to question 3** (Do you think English language learning is good or beneficial for you? Why? Why not?)

Most of the participants considered learning English very beneficial, especially for future work (84.3%) and continuing education (76.5). The other reasons that they mentioned for the benefits of learning this language included the ability of communication with people from all over the world (87.5%), social status and prestige that knowing this foreign language can give to a person in Erbil (57.8%), watching original movies in English (45.3%), and the last one was travelling and studying abroad (67.1%).

To mention some of the responses and the participants’ elaboration on their answers we can refer to the following responses:

“I think it’s very useful especially because we live in a new world. English is the international language and if we know it we can use it everywhere, we can communicate, trade and study everywhere with different people from countries other than ours.”

“Of course, it is important and beneficial. It is the international language and the main language of the world. We live in the era of technology. Working with computers, internet, and working with different programs mostly need knowing English and if we know it well it can be really helpful. It makes finding a good job easy and we can watch our favorite English language movies and read original books easily in English and do not need to look for their translations or subtitles of the films in our own language.”

**Learners’ responses to question 4** (What do you think about English language and its instruction at school?)

Majority of the participants had positive attitudes towards English as a foreign and international language (96.8%), and most of them also considered it crucial to be included in school curriculum and be taught as one of main subjects (93.75). To mention some of the responses and the participants’ elaboration on their answers we can refer to the following responses:

“I think English is a very sweet language, and its learning is easy if it is taught well at school.”

“I feel learning English gives me self-confidence and power. I like to become fluent in English language speaking and use it easily. But I feel it’s not being taught well at schools. For example, in our school English teachers mostly concentrate on grammar rather than conversation.”

“English language is not my field of interest, but I study this school subject a lot and I want to learn it well because it is one of our main school subjects and the score, I get for this class is important both for me and for my family.”

**Learners’ responses to question 5** (To what extent do you think your teacher was effective in motivating you?)

The majority of the participants (9 3.7%) considered the teacher’s role very effective in motivating them. To mention some of the responses and the participants’ elaboration on their answers we can refer to the following responses:

“Our teacher has a great role in this respect. Especially due to the interesting class activities that he assigns to us and the out-of-class activities. For example, he asked us to search in the internet and collect information about a specific subject. Activities like making wall newspaper. His teaching practice and the way of his teaching is so interesting and attractive to me.”

“I really like my English teacher. One of the major reasons for my motivation in learning English is my teacher. Before previous years, I was weak in the English language and I even felt that I did not like studying English at all. But this year, my English teacher made me attracted in learning this language. Especially with his specific behavior and the attention he paid to me in English class, he gave me self-confidence and raised my self-esteem. He always encouraged me and did not look at me as a weak student in class, that’s why I became encouraged and motivated to study more, and now I like to study more to make my teacher and my family more satisfied with me.”

**Learners’ responses to question 6** [To what extent do you think the educational system was effective in motivating you (i.e., in terms of the educational syllabus, material, or books)].

Most of the students who participated in this study considered the role of the educational system very important and effective in motivating the students (98.4%). To mention some of the responses and the participants’ elaboration on their answers we can refer to the following responses:

“I think what is being taught at school has been effective in creating motivation in me. For example, our English textbook is very good. Nearly all needed contents are included in this book. It is attractive to some extent. I think we have a good educational system because overall, school is the main motivating factor in me.”

## Discussion

The answers to the research questions of this study are represented and discussed below in light of the obtained results that were elaborated in the previous section.

Considering the first main research question of this study “Is there any difference between male and female Kurdish students’ beliefs and perceptions towards their own motivational level, type of motivation, and the effectiveness of the motivational strategies used by their teachers*?*” the results showed that there was no significant difference between male and female Kurdish students’ beliefs and perceptions towards their own motivational level, type of motivation, and the effectiveness of the motivational strategies used by their teachers. Based on the findings of this study, majority of Kurdish students were interested in learning English language as a foreign language and they mostly considered themselves motivated in learning this language. They also mostly preferred to learn English for itself, not as a class requirement. It must be asserted that no significant difference was observed in the responses of the male and female students. we should refer to the results which showed that in general all Kurdish EFL learners in our study mostly had both instrumental and integrative orientations towards learning English. However, female students showed to be more instrumentally motivated than male students, while for male students, integrative orientation was much higher than being instrumentally motivated. They had integrative motivations due to the fact that they mostly loved learning English for they considered it important for facilitating the communication and interaction with foreigners. They mostly have strong desire to learn the target language and to become fluent in that. On the other hand, they showed to be instrumentally motivated since they were mostly motivated to learn foreign language for the reasons such as getting a good job and becoming more educated, and also due to the fear of getting bad mark or disappointing their family and culture.

Regarding the research question “*What is the Kurdish EFL students’ attitude towards English language and its instruction at school?*,” the results of this study showed that generally, Kurdish EFL students (both male and female) had positive attitudes and perceptions towards learning English as a foreign language. In fact, they generally loved English language and had overall favorable attitudes regarding learning this language as a foreign and international language, and most of them considered it very important to be included in school curriculum and be taught as one of main school subjects.

The other related findings also showed that both male and female Kurdish learners of English language had positive attitudes towards native English speakers and communicating with native speakers of English was interesting to them. Moreover, considering learners’ attitude and beliefs about their English classes, most of Kurdish learners who participated in this study showed their love and interest regarding English subject, however they mostly declared that their class atmosphere was not enjoyable enough and it is somehow boring.

Exploring the attitudes and beliefs of male and female Kurdish EFL students also showed that learning English is very beneficial to them, especially for their future work and continuing education, increasing the communication with people from all over the world, the social status and prestige that knowing this foreign language can give to a person in Erbil, watching original movies in English, and the travelling and studying abroad. Considering the role of teacher in motivating the students from the Kurdish EFL learners’ perspective, it was found that while they (both male and female learners) generally believed that teacher’s role is very effective in motivating and demotivating the students, majority of these students did not find their own teachers’ role and practice much effective. In fact, they mostly believed that their teachers were generally good and inspirational majority of Kurdish EFL students (both male and female) considered educational system as being highly effective and believed that it plays a very important role in motivating the students in learning English language.

Other findings of this study based on Kurdish EFL students’ opinions on the related issues which are interesting and noteworthy indicate that Majority of Kurdish students believe their parents are good motivators and have special role in directing and encouraging them towards learning English as a foreign language, and they mostly considered their parents as being actually supportive enough in helping them with their studies and pleasing them is very important for the students. Moreover, better future job opportunities, more future academic success, educational setting, class atmosphere, teaching aids and learning facilities, teachers along with their behaviors and methods or ways of teaching, are found to be the most frequent factors which the Kurdish EFL students perceive as being effective in motivating or demotivating them.

Concerning the second main research question *“*Is there any difference between the L2 motivational system components (i.e., ideal L2 self, ought-to L2 self, L2 learning experience) which mostly affect male and female Kurdish students’ motivation and language proficiency?*”* based on the results obtained from the Kurdish EFL learners’ responses to the L2MSS questionnaire, It was found that generally all three main components of L2 motivational self-system (i.e., ideal L2 self, ought-to L2 self, L2 learning experience) highly affect both male and female students’ motivation almost similarly and consequently affect their language proficiency. Considering other motivating factors that was assessed through questionnaire, it was also found that family and culture, positive attitude towards learning English, and criterion measure that represents students’ extent of interest in English language class play a great role in motivating both female and male students. Both male and female students did not show much fear of assimilation, which is good, because having fear of assimilation might make the learners less motivated and make them show avoidance behavior. However, both male and female students did not consider the culture of their English language classes interesting and motivating, and both groups showed high range of English language anxiety, while the English anxiety of female students were slightly higher than male students. Therefore, special attention must be directed towards improving the motivating factors and removing demotivating factors by teachers and the education system.

All in all, considering the EFL students’ level of motivation, the results of this study are in line with results of the studies by Ditual (2012), Özütürk and Hürsen (2014), Al-Sohbani (2015), in that the EFL students in all of these research works along with the participants of our work showed to have high levels of English language learning motivation.

This study also provides support for the studies by Ditual (2012), Zanghar (2012), and Kashefian-Naeeini et al. (2018), in that EFL students generally have both types of integrative and instrumental motivation towards language learning. However unlike the findings of Zanghar (2012) and Kashefian-Naeeini et al. (2018), and similar to the findings of the results of this study revealed that although both types of integrative and instrumental motivations have been observed in the students, the amount of the integrative type of motivation is highly greater than their instrumental orientation towards learning English language. Moreover, unlike the findings of Rezaee et al. (2015), and similar to the findings of Lucas (2010), it has been found that the students mostly have intrinsic type of motivation, although both intrinsic and extrinsic types of motivation are observed among the participants.

The findings of this study regarding factors affecting the students’ motivation are also consistent with the findings of Fatiha et al. (2014), Rostami et al. (2015), and Yurtseven et al. (2015), in that all of them highlighted the important role of teachers, the methodologies and motivational strategies they adopt and use in their classes, providing enjoyable learning atmosphere, and also their attitudes and behaviors on motivating the students, along with other factors. The findings of this study are also in line with the Tilya (2012).

Who put emphasize on the positive and most effective role of teachers on enhancing students language learning motivation.

The findings of this study are also in line with the findings of Özütürk and Hürsen (2014), Al-Sohbani (2015), Kashefian-Naeeini et al. (2018), and Hussein and Bajalani (2019), which showed that EFL students generally have positive and favorable attitudes towards English language and its learning as a foreign language, while our study’s results are in contrary to the findings of Gabillon (2007) that revealed negative attitudes of EFL learners towards English language learning.

Considering the effectiveness of L2 motivational self-system components and its relationship with students’ motivation, the findings of this study provide support for the findings of the studies by Zoabi (2012), Magid (2014), Moskovsky et al. (2016), and Sherafati and Ghafournia (2019), in that it is found a positive relationship between L2 motivational self-system and EFL students’ motivation towards learning English as a foreign language, and also in that l2 motivational self-system components are predictive of students’ English language learning motivation. However, the findings of this study are in contrary to the findings of the studies by Papi and Abdollahzadeh (2012), Mac Intyre and Serroul (2015), and Moskovsky et al. (2016), which have the findings opposite to the findings of this study in this respect.

### Implications

The results of this study has provided many useful information about Kurdish students’ extent of English language learning motivation and the factors that have impact in motivating and demotivating them by the declaration, experience and beliefs of the participants of this study. All of these information has implications for the teachers, policy makers, educators and all involved in the education system. In general, to provide better learning environment and try to find the best strategies and policies to enhance students’ English language learning motivation and consequently increase their language learning achievement in general.

## Conclusion

The fact that in this study we found majority of Kurdish EFL students are highly motivated by having both instrumental and integrative motivation is very promising, which shows that education system in this region of Iraq can be hopeful about the future of their students and the effectiveness of their system of education in general. Moreover, teachers can take advantage of the finding which shows the students’ integrative motivation is higher than their instrumental motivation, and try to choose and plan appropriate strategies and ways to help them also increasing their instrumental motivation.

The most frequent motivational strategies and ways of motivating used and recommended by the teachers in this study are also helpful, especially for teacher educators in teacher training programs, so that they can get aware of the mostly used strategies by Kurdish EFL teachers, and try to improve and correct them, and try to teach them the most effective strategies and ways of raising motivation in the students.

Generally, in the light of the main findings of the study, it can be concluded that special attention must be paid to the students’ motivation for the important and crucial role that it plays not only on the students’ educational achievements and success, but also on their future job opportunities and social status. At the end, it is hoped that the outcomes of this intensive research work be useful in developing the students’ English language learning motivation and in helping the education system and the teachers in setting appropriate plans and strategies to make that happen.

## Data availability statement

The datasets presented in this study can be found in online repositories. The names of the repository/repositories and accession number(s) can be found in the article/Supplementary material.

## Ethical statement

The studies involving human participants were reviewed and approved by Ethical Committee Board of Cyprus International University. The patients/participants provided their written informed consent to participate in this study.

## Author contributions

All authors listed have made a substantial, direct, and intellectual contribution to the work and approved it for publication.

## Conflict of interest

The authors declare that the research was conducted in the absence of any commercial or financial relationships that could be construed as a potential conflict of interest.

## Publisher’s note

All claims expressed in this article are solely those of the authors and do not necessarily represent those of their affiliated organizations, or those of the publisher, the editors and the reviewers. Any product that may be evaluated in this article, or claim that may be made by its manufacturer, is not guaranteed or endorsed by the publisher.
