# Supplementary material for: Investigation of Kurdish students’ L2 motivational self-system and their motivational beliefs in high school
Source: Front Psychol. 2022 Oct 25;13:974748. doi: 10.3389/fpsyg.2022.974748 (PMC9642068; doi:10.3389/fpsyg.2022.974748)
Supplement: Supplementary file 1 [file Table_1.DOCX]

**Appendix**

**Learners’ questionnaire**

(Adapted from L2MSS questionnaire of Dornyei et al. (2006))

**Consent Form**

Dear student,

I invite you to participate in the study that is planned to understand the sources of your motivation. The study is titled “An exploratory Study on Second Language Learners’ Sources of Motivation and Teachers’ Motivational Strategies in High Schools”. In the attached questionnaire, there are 41 items.

If you agree to participate in this study, please complete the questionnaire that is attached to this letter. There are not any correct or incorrect answers. Please complete the questionnaire based on your experiences as English language learners. Your answers will help me to improve your language learning experiences at school.

You can contact me if you have any further questions regarding your participation in my research.

**The Background Questions**

Gender: Male Female

Age:

Year of study:

City:

**English Learner Questionnaire**

In this part, we would like you to tell us how much you agree or disagree with the following

| 1 | | 2 | 3 | | 4 5 6 | | |  |  |
| --- | --- | --- | --- | --- | --- | --- | --- | --- | --- |
| Strongly  disagree | Disagree | | | Slightly  Disagree | | Slightly Agree  Agree | Strongly  Agree | | |

statements by simply circling a number from 1 to 6. Please do not leave out any items.

(Example) If you slightly disagree with the following statement, write this:

I like skiing very much. 1 2 3 4 5 6

| Strongly agree | Disagree | Slightly Disagree | Slightly Agree | Agree | Strongly Agree |
| --- | --- | --- | --- | --- | --- |

| No. | Item | 1 | 2 | 3 | 4 | 5 | 6 |
| --- | --- | --- | --- | --- | --- | --- | --- |
| 1 | If an English course was offered in the future, I would like to take it. |  |  |  |  |  |  |
| 2 | If my teacher would give the class an optional assignment, I would certainly |  |  |  |  |  |  |
| 3 | I would like to study English even if I were not required. |  |  |  |  |  |  |
| 4 | I would like to concentrate on studying English more than any other topic. |  |  |  |  |  |  |
| 5 | I am prepared to expend a lot of effort in learning English. |  |  |  |  |  |  |
| 6 | I am working hard at learning English. |  |  |  |  |  |  |
| 7 | I can imagine myself studying in a university where all my courses are taught in English. |  |  |  |  |  |  |
| 8 | Whenever I think of my future career, I imagine myself using English. |  |  |  |  |  |  |
| 9 | I can imagine myself speaking English with international friends or colleagues. |  |  |  |  |  |  |
| 10 | I can imagine myself living abroad and using English effectively for communicating with the locals. |  |  |  |  |  |  |
| 11 | I can imagine myself speaking English as if I were a native speaker of English. |  |  |  |  |  |  |
| 12 | I can imagine myself writing English e-mails/letters fluently. |  |  |  |  |  |  |
| 13 | I study English because close friends of mine think it is important. |  |  |  |  |  |  |
| 14 | Learning English is necessary because people surrounding me expect me to do so. |  |  |  |  |  |  |
| 15 | I consider learning English important because the people who respect me think that I should do it. |  |  |  |  |  |  |
| 16 | If I fail to learn English, I’ll be letting other people down. |  |  |  |  |  |  |
| 17 | Studying English is important to me in order to gain the approval of my peers/teachers/family/and boss. |  |  |  |  |  |  |
| 18 | Studying English is important to me because other people will respect me more if I have a knowledge of English. |  |  |  |  |  |  |
| 19 | My parents encourage me to practice my English as much as possible. |  |  |  |  |  |  |
| 20 | My family put a lot of pressure on me to study English. |  |  |  |  |  |  |
| 21 | My parents/family believe(s) that I must study English to be an educated person. |  |  |  |  |  |  |
| 22 | Studying English is important to me in order to bring honor to my family. |  |  |  |  |  |  |
| 23 | Being successful in English is important to me so that I can please my parents/relatives. |  |  |  |  |  |  |
| 24 | I have to study English, because, if I don’t do it, my parents will be disappointed with me. |  |  |  |  |  |  |
| 25 | Do you like the atmosphere of your English classes? |  |  |  |  |  |  |
| 26 | Do you always look forward to English classes? |  |  |  |  |  |  |
| 27 | Do you find learning English really interesting? |  |  |  |  |  |  |
| 28 | Do you really enjoy learning English? |  |  |  |  |  |  |
| 29 | Do you think time passes faster while studying English? |  |  |  |  |  |  |
| 30 | Would you like to have more English lessons at school? |  |  |  |  |  |  |
| 31 | I think that there is a danger that Iranian people may forget the importance of Kurdish culture, as a result of internationalization. |  |  |  |  |  |  |
| 32 | Because of the influence of the English language, I think the Kurdish language is corrupt. |  |  |  |  |  |  |
| 33 | Because of the influence of the English-speaking countries, I think the morals of Kurdish people are becoming worse. |  |  |  |  |  |  |
| 34 | I think the cultural and artistic values of English are going at the expense of Kurdish values. |  |  |  |  |  |  |
| 35 | I think that, as internationalization advances, there is a danger of losing the Kurdish identity. |  |  |  |  |  |  |
| 36 | How nervous and confused do you get when you are speaking in your English class? |  |  |  |  |  |  |
| 37 | How afraid are you that other students will laugh at you when you speak English? |  |  |  |  |  |  |
| 38 | How uneasy would you feel speaking English with a native speaker? |  |  |  |  |  |  |
| 39 | How tense would you get if a foreigner asked you for directions in English? |  |  |  |  |  |  |
| 40 | How afraid are you of sounding stupid in English because of the mistakes you make? |  |  |  |  |  |  |
| 41 | How worried are you that other speakers of English would find your English strange? |  |  |  |  |  |  |

**Figure 1.**

**Figure 2.**
